# Association of Serum Lipids and Obstructive Lung Disease in Hispanic/Latino Adults of Diverse Backgrounds

**DOI:** 10.4172/2161-105X.1000419

**Published:** 2017-08-31

**Authors:** Majid Afshar, Donghong Wu, Ramon Durazo-Arvizu, Frank G Aguilar, Ravi Kalhan, Sonia M Davis, Robert Kaplan, Oana L Klein, Eliana P Mende, Maria S Pattany, Martha L Daviglus

**Affiliations:** 1 Division of Pulmonary and Critical Care, Loyola University Stritch School of Medicine; 2 Institute for Minority Health Research, University of Illinois at Chicago; 3 Department of Public Health Sciences, Loyola University Stritch School of Medicine; 4 Department of Medicine, University of Illinois at Chicago College of Medicine; 5 Division of Pulmonary and Critical Care, Northwestern Feinberg School of Medicine; 6 Department of Biostatistics, University of North Carolina Chapel Hill; 7 Department of Epidemiology and Population Health, Albert Einstein College of Medicine; 8 Department of Medicine, University of California San Francisco School of Medicine; 9 Division of Pulmonary and Critical Care, University of Miami School of Medicine; 10 Division of Pulmonary and Critical Care, University of Miami Behavioral Medicine Research Center

**Keywords:** Obstructive lung disease, Asthma, Chronic obstructive lung disease, Lipids, Hispanic

## Abstract

**Rationale:**

Substantial variation in the prevalences of obstructive lung disease exist between Hispanic/Latino heritage groups. Experimental studies have posited biological mechanisms linking serum lipids and lipid-lowering medications with obstructive lung disease. The aim of this study is to examine the associations of serum lipid levels with the prevalences of asthma and chronic obstructive pulmonary disease in the Hispanic Community Health Study/Study of Latinos and how these associations vary by Hispanic/Latino heritage group.

**Methods:**

The Hispanic Community Health Study/Study of Latinos is a population-based probability sample of 16,415 self-identified Hispanic/Latino persons aged 18–74 years recruited between 2008 and 2011 from randomly selected households in four US field centers. The baseline clinical examination included comprehensive biological testing (fasting serum lipid levels), behavioral and socio-demographic assessments, medication inventory including inhalers, and respiratory data including questionnaires for asthma and standardized spirometry with post-bronchodilator measures for identification of obstructive lung disease.

**Measurements and main results:**

Hispanic/Latinos with current asthma had lower age- and statin-use-adjusted mean serum total cholesterol, low-density lipoprotein cholesterol, and triglyceride levels than their non-asthmatic counterparts. In analysis adjusted for age plus gender, ethnicity, cigarette smoking, alcohol intake, body mass index, lipid/cholesterol-lowering medications, age at immigration, health insurance status, and use of oral corticosteroids, increasing serum levels of total cholesterol and low-density lipoprotein cholesterol were associated with lower odds of current asthma in the estimated population. Unlike asthma, Hispanic/Latinos with chronic obstructive pulmonary disease had lower mean high-density lipoprotein than their non- chronic obstructive pulmonary disease counterparts. In the fully adjusted analysis no significant associations were found between lipid levels and prevalent chronic obstructive pulmonary disease.

**Conclusions:**

We showed a modest inverse relationship between serum lipid levels and current asthma. These results highlight some important differences in Hispanics/Latinos and certain serum lipids may be factors or markers of obstructive lung disease.

## Introduction

Hispanics/Latinos comprised 16% of the United States (U.S.) population with over 50 million in 2010 and are the fastest growing ethnic minority group [1]. Obstructive lung disease, including chronic obstructive pulmonary disease (COPD) and asthma, is the third leading cause of death in the U.S [2]. Substantial variation in the prevalences of asthma and COPD exist between Hispanic/Latino heritage groups [3–5]. In U.S. population surveys, the prevalence of asthma was highest in U.S. Hispanics with Puerto Rican heritage and lowest in those with Mexican heritage, with non-Hispanic whites and blacks in between [6–8]. In addition, age-adjusted mortality rates for COPD in the U.S. are reported to be the highest in Puerto Ricans (15.5 per 100,000 individuals) and the lowest in Mexicans (6.4 per 100,000 individuals) [7]. A prior study from the Hispanic Community Health Study/Study of Latinos (HCHS/SOL) was among the first to establish the prevalences of both diseases in multiple Hispanic/Latino heritage groups in the U.S. and helped elucidate important factors for varying prevalences of asthma and COPD [9]. Additional studies are needed to further clarify the disparities in prevalence in this heterogeneous population.

Experimental studies have posited biological mechanisms linking serum lipids and lipid-lowering medications with respiratory diseases, especially COPD [10–13]. In murine studies, disruption of triglyceride and cholesterol metabolism was shown to induce respiratory inflammation and emphysema [11,12]. However, clinical trials investigating the use of statins in COPD have been equivocal [14]. Observational studies performed in individuals with asthma have not added much clarity to the role of lipids and cholesterol-lowering medications in obstructive lung disease [15–17]. Studies have demonstrated positive, negative, and null associations between dyslipidemia and asthma [17–20]. While smaller observational studies have shown a positive association between lipids levels and poor pulmonary function, a larger study from the National Health and Nutrition Examination Survey (NHANES) showed an inverse association, especially in Mexican Americans [21]. These disparate results may be partially explained by variation in lipid profiles across heritage groups, suggesting obstructive lung disease may also vary between individuals in the U.S. from different Hispanic/Latino heritages. Although prior studies have established the lipid profile in Hispanic/Latino heritage groups [22,23], the association with obstructive lung disease has not been examined.

Examinations in health risk factors among Hispanic/Latino heritage groups are complex and cannot be generalized because of variations in genetic ancestry, socioeconomic status, acculturation, environmental exposures, and health status [24]. HCHS/SOL with its large, diverse Hispanic/Latino population provides the opportunity to investigate the association of serum lipid levels with asthma and COPD between multiple Hispanic/Latino heritages. Our hypothesis is the associations of serum lipid levels with the prevalences of asthma and COPD will be positive in HCHS/SOL, and that these associations will vary by Hispanic/Latino heritage groups.

## Methods

HCHS/SOL is a community-based prospective cohort study designed to estimate the prevalence of chronic diseases and examine the relationships between risk factors and outcomes in a diverse Hispanic/Latino population comprised of Mexican, Puerto Rican, Cuban, Dominican, Central American, and South American people living in the U.S. Details of the sampling methods and study design were previously published [25,26]. The analysis for this study only incorporates data from the baseline visit and is cross-sectional. Briefly, between 2008 and 2011, the HCHS/SOL recruited self-identified Hispanics/Latinos aged 18–74 years from randomly selected households in four urban communities in the U.S. (Bronx, New York; Chicago, Illinois; Miami, Florida; San Diego, California). Households were selected using a stratified, two-stage area probability sample of addresses selected in each of the four field centers with oversampling in areas of Hispanic/Latino concentration and persons aged 45–74 years to facilitate examination of targeted outcomes [22]. Sampling weights were generated to reflect the probabilities of selection at each stage. Participants included in this analysis had data on all of the following parameters: serum lipid levels including total cholesterol (TC), high-density lipoprotein cholesterol (HDL-C), low-density lipoprotein cholesterol (LDL-C), and triglycerides (TGs); pre- and post-bronchodilator spirometry; characteristics and comorbidities related to cardiac and pulmonary health; medication use; and self-report questionnaire data on asthma and COPD. The study was approved by Institutional Review Boards at the data coordinating center and each field center where subjects gave written consent.

### Survey response rate

The household-level response rate of the identified households in the survey design was 33.5%. Of 39,384 individuals who were selected, screened and met eligibility criteria, 41.7% were enrolled, representing 16,415 persons from 9,872 households. Even though the response rate is low, a widely accepted statistical adjustment protocol was followed to reduce the potential bias of estimates due to study non-participation. To minimize this bias effect while controlling the precision loss implications of adjustment, the sample weight of each participant was: (1) calculated based on its selection probability; (2) adjusted for differential non-response at the household and person levels, and trimmed to reduce the variability of the adjusted weights; and (3) calibrated to the 2010 U.S. Census count by age, gender, and Hispanic background in each field center’s target population. This three-step approach to calculate sample weights is consistent with weighting strategies used in other major health surveys utilizing probability sampling.

### Data collection

Data were collected during a baseline examination conducted at each field site and included biological measures, interviewer-administered questionnaires, and medication inventory. Heritage was assessed via the question, “Which best describes your Hispanic/Latino heritage?” The mixed/other Hispanic/Latino group includes those participants of mixed heritage groups and also those participants who did not identify belonging to a specific heritage group. Participants were asked to fast and refrain from smoking for twelve hours prior to their examination and to avoid vigorous physical activity the morning of the visit. Participants were instructed to bring all prescription and nonprescription medications taken in the past month to the study center at the time of their physical examination. Questions were also translated into Spanish and confirmed in focus groups.

### Cholesterol measurements

Serum TC was measured using a cholesterol oxidase enzymatic method and HDL-C with a direct magnesium/dextran sulfate method. LDL-C was calculated using the Friedewald equation [27]. Additional measurements of lipid levels included TGs. Dyslipidemia was defined as TC of 240 mg/dL or greater, LDL-C of 160 mg/dL or greater, HDL-C of less than 40 mg/dL, or currently taking cholesterol/lipid-lowering medications [28]. Use of cholesterol/lipid-lowering medications was established by an affirmative response to the question, “Were any of the medications you took during the last four weeks for high blood cholesterol?” or confirmation during the medication inventory.

### Asthma identification

Self-reported current asthma was assessed via a previously published respiratory questionnaire [29]. Current asthma was defined as an affirmative answer to the following questions: (1) Do you still have it [asthma]?” (2) Was it [asthma] diagnosed by a doctor or other health professional?” Use of asthma medications was established by an affirmative response to the question: “Were any of the medications you took during the last four weeks for asthma?” or confirmation during the medication inventory.

### COPD identification

Spirometry was conducted in accordance with the American Thoracic Society/European Respiratory Society guidelines [30]. Automated quality checks following another National Heart, Lung, and Blood Institute protocol [31] with over-reading by one investigator performed using a dry-rolling-sealed spirometer. Participants with a pre-bronchodilator forced expiratory volume in one second/forced vital capacity (FEV_1_/FVC) less than or equal to 0.70 or less than the lower limit of normal [32] met criteria for post-bronchodilator spirometry. Current COPD was defined by a post-bronchodilator FEV_1_/FVC ratio of 0.70 or less and is in accordance with the 2011 American Thoracic Society/European Respiratory Society and 2017 Global Initiative for Chronic Obstructive Lung Disease clinical practice guidelines. The percent predicted values was calculated by the FEV1 (mL) and FVC post-bronchodilator measurement divided by the predicted FEV1 (mL) and FVC using the National Health and Nutrition Examination Survey III reference equations. Because COPD includes bronchitis and emphysema, use of COPD medications was established by an affirmative response to the question: “Were any of the medications you took during the last four weeks for chronic bronchitis or emphysema?” or during medication inventory.

### Additional variables

Height and weight were measured using a standardized protocol. Body mass index (BMI) was calculated as weight in kilograms divided by height in meters squared [33]. Current smokers were defined by an affirmative response to the question: “Do you now smoke daily, some days, or not at all?” Former cigarette smokers were defined by an affirmative response to the question: “Have you ever smoked at least 100 cigarettes in your entire life?” Current alcohol users were defined by an affirmative response to the question: “Do you presently drink alcoholic beverages?” Former alcohol users were defined by an affirmative response to the question: “Did you ever drink alcohol?” and a negative response to the question: “Do you presently drink alcoholic beverages?” Age of immigration to the mainland U.S. was defined as current age minus number of years living in the U.S., assessed by the item: “From the time that you first moved to the U.S. to today, about how many years have you lived in the mainland U.S. (mainland 50 states and D.C.)?” Individuals born in the mainland U.S. received a 0 for age at immigration. Atopy was defined as affirmative answers to: “Have you ever had any hay fever allergy involving the nose and/or eyes?” and “In the past 12 months, have you received medication, taken medications or used a nasal spray for hay fever?”

## Statistical analysis

Descriptive statistics were age-adjusted and computed for all individuals by Hispanic/Latino heritage group. Estimates are applicable to the study target population, defined as Hispanics/Latinos ages 18–74 years living in the four study communities. Prevalences of asthma and COPD were estimated for the population and within each Hispanic/Latino heritage group. Mean serum lipid levels in the asthma and COPD heritage groups were age and statin-use adjusted. For categorical responses, age-adjusted odds ratios (OR) for the estimated population of Hispanic/Latinos in the four HCHS/SOL communities were calculated using survey logistic regression. Multivariable logistic regression was used to model the associations of lipid levels with current asthma and COPD. All variables in the adjusted model for asthma and COPD were chosen a priori as risk factors or confounders for these diseases [7,9,34–38]. Both oral and inhalation/intranasal glucocorticoid use were previously shown to increase HDL-C among individuals above 60 years of age [39]. Therefore, oral glucocorticoid use was included in the adjusted analysis. The following models were performed in the analyses: unadjusted (model 1); adjusted for age only (model 2); adjusted for age plus gender, ethnicity, cigarette smoking, alcohol intake, BMI, lipid/cholesterol-lowering medications, age at immigration, health insurance status, and use of oral corticosteroids (model 3). The COPD model 3 additionally adjusted for asthma onset before age 45.

Missing assessment of COPD was accounted for using a three-step process identical to previous methods published from HCHS/SOL [9]. This is to address the potential impact the missing spirometry data may have on COPD prevalence as described in Supplemental. Inverse probability weighting was used to account for missing pre-bronchodilator spirometry data. Weights were developed using a logistic regression model from a set of baseline covariates. In Step 1, the missing covariates that were needed in subsequent inverse probability weight (IPW) calculation were imputed by multiple imputation. In Step 2, a logistic regression was fit to predict the missing status of the first spirometry. The inverse probability weightings (IPW) were calculated using the estimated probability of missing first spirometry from the regression. In step 3, COPD status for participants with missing post-bronchodilator spirometry was then imputed with multiple imputation. For the multiple imputation step, five imputed datasets were generated and the estimates were combined by Rubin’s rule. The COPD prevalences were estimated by survey logistic regression from step 3 and the sample weight (the original sampling weight times IPW weight from step 2). All analyses were performed using SAS version 9.4 (SAS Institute, Cary, NC).

## Results

Of the 16,415 persons from 9,872 households in HCHS/SOL, 1779 (10.8%) were excluded from this analysis for missing laboratory data (n=497), missing asthma status (n=407), and missing demographic or covariate responses (n=875). Multiple imputations and inverse probability weighting were used for missing spirometry data as previously described. Individuals excluded because of missing demographic and covariate responses comprised 6.0% of the population but in the unadjusted and age-adjusted logistic regression analysis for the association between serum lipid parameters and current asthma, the odds ratios and 95% CIs were similar with and without inclusion of this group (data not shown).

Participants that completed the respiratory questionnaire and provided a blood sample for baseline data on lipid components accounted for 88.5% (n=14,636) of those screened and enrolled. The sample with COPD data was 14,012 (85%). The estimated Hispanic heritage in the four communities based on this analysis sample was greatest for Mexicans at 37.7% followed by Cubans (20.9%), Puerto Ricans (15.3%), Dominicans (9.4%), Central Americans (7.8%), South Americans (5.0%), and Mixed/Other (4.1%).

### Estimated population characteristics

A majority of the estimated population in HCHS/SOL was never smokers and over half were current alcohol drinkers. Puerto Ricans had the highest prevalence of current smoking and the lowest prevalence of never smoking amongst all Hispanic/Latino groups (p<0.001). Among the current smokers, Cubans had the highest proportion of individuals with greater than 10 pack-years of smoking (p<0.001). These results are displayed in Table 1.

The overall age-adjusted prevalence of dyslipidemia was 29.3% with 7.8% of the estimated population taking statin medications. Central Americans had the highest mean serum levels of TC, LDL-C, and TGs, and the greatest prevalence with dyslipidemia. Dominicans had the lowest prevalence of dyslipidemia and the lowest mean serum level of TGs. The use of statin medications was highest in Puerto Ricans.

The overall age-adjusted prevalence of current asthma without COPD was 7.4% (95% CI 6.7–8.0) in the estimated population. Among those with current asthma without COPD, 32.0% (95% CI 27.7–36.3) had atopy, 25.6% (95% CI 21.8–29.3) were current smokers, and 57.4% (95% CI 53.1%–61.7%) had a BMI of 30 or greater (Table 2). The prevalence of current asthma was highest in Puerto Ricans at 20.7% and lowest in South Americans, Central Americans, and Mexicans (range 3.3–3.5%). Mean serum lipid levels in the estimated population with current asthma varied by Hispanic/Latino heritage group. Age-statin use-adjusted mean serum TC, LDL, and TG were lowest in Puerto Ricans, Dominicans, and South Americans compared to their counterparts in the other Hispanic/Latino heritage groups (Table 3).

The prevalence of COPD in the age-adjusted estimated population of HCHS/SOL was 3.6% (95% CI 3.2–4.0). Puerto Ricans and Cubans had the highest prevalence of COPD between Hispanic heritage groups. Over a third of the estimated population with COPD only were never smokers, and nearly 20% had dyslipidemia but only 2.3% (95% CI 0.3–4.4) reported taking inhaler medications and 3.7% (95% CI 1.2–8.6) reported taking statin medications (Table 2). The estimated population with both current asthma and COPD had the highest prevalences for inhaler medication and oral glucocorticoid use. In this group, prevalences of those born in the US, health insurance status, and using statin and inhaler medications was greater than the COPD only group and similar to the asthma only group (Table 2).

### Association of serum lipid levels with current asthma and COPD

Hispanic/Latinos with current asthma had lower age- and statin-use-adjusted mean serum TC, LDL-C, and TG levels than their non-asthmatic counterparts (Table 3). In analysis adjusted for age plus gender, ethnicity, cigarette smoking, alcohol intake, BMI, lipid/cholesterol-lowering medications, age at immigration, health insurance status, and use of oral corticosteroids, increasing serum levels of TC and LDL-C were associated with lower odds of prevalent asthma in the HCHS/SOL population. The association was strongest in LDL-C with an OR 0.86 (95% CI 0.76–0.98) per each 50 mg/dL increase. The Dominican heritage group demonstrated a lower OR for prevalent asthma per each 50 mg/dL higher serum LDL-C and TC levels (Table 4). However, no interaction was found between LDL-C and heritage group (p=0.88) nor TC and heritage group (p=0.78). Unlike asthma, Hispanic/Latinos with COPD had lower mean HDL than their non-COPD counterparts (Table 5). In the fully adjusted analysis no significant associations were found between lipid levels and prevalent COPD (Table 6). In examination of heritage groups, Central Americans had a higher OR of 1.45 (95% CI 1.17–1.79) for COPD prevalence per each 10 mg/dL increase in HDL. However, no interaction was demonstrated between heritage group and HDL-C (p-value=0.31) in the fully adjusted model.

## Discussion

Our study is the first nationally representative study of a diverse group of Hispanic/Latinos and their association between lipid levels and obstructive lung disease. We found a decreased odds risk association between current asthma and both TC and LDL-C. Conversely, no significant associations were found between serum lipid levels and COPD in the estimated HCHS/SOL population.

HCHS/SOL is amongst the few epidemiologic studies that have examined the role of lipids in asthma prevalence, especially between various Hispanic/Latino groups. Prevalence at 7.6% for current asthma in HCHS/SOL was similar to the general U.S. population [8]. This similarity occurred in spite of a greater proportion with asthma risk factors such as obesity and cigarette smoking [34–36,40–42]. The prevalence for COPD was lower than the general U.S. population [8,43] 6.3% for U.S. adults in 2011 [44] compared to 3.6% for our HCHS/SOL estimated population. Differences may have occurred because the average age in our estimated population was 41.2 years of age whereas other studies from HCHS/SOL showed a higher prevalence rate of 8.2% by examining only participants over 45 years old [9]. Of note, over one-third of the estimated population with COPD were never-smokers which is higher than other nationally representative health surveys and epidemiologic studies [45–47]. This disparity supports the need to more closely examine the Hispanic/Latino group and possibly examine other risk factors such as environmental and occupational exposures that may be specific to this group [48]. Furthermore, the prevalences in the estimated population with asthma had more insurance coverage, more born in the US, and more taking cholesterol and inhaler medications than in the COPD group possibly reflective of a group more engaged with the U.S. healthcare system. Not only did prevalence of respiratory disease vary substantially between Hispanic/Latino groups, but prevalence of cardiovascular disease risk factors varied as well [6,22,37]. Nearly 30% of all Hispanic/Latinos had dyslipidemia in the HCHS/SOL sampled population which was higher than published population estimates ranging between 12 and 24% in non-Hispanic whites [23,49–51].

Variations of asthma prevalence in different heritages of Hispanic/Latinos have been attributed to racial ancestry (beyond trunk-to-limb ratio), healthcare access, genetic susceptibility [52,53], and environmental exposures [6]. In addition, other previously described risk factors in the general population such as atopy, obesity, smoking was encountered in one-quarter to one half of the estimated population with current asthma in HCHS/SOL [36,41,42]. Acculturation and earlier age of immigration to the U.S. was also previously associated with higher asthma prevalence in HCHS/SOL [9,54]. In regards to the role of lipids, only one study using data from the National Health and Nutrition Examination Survey previously demonstrated an inverse association between serum lipid levels and asthma, specifically in Mexican-Americans [21]. After accounting for multiple asthma risk factors, we found the estimated Hispanic/Latino population had significant but small associations between TC and asthma as well as LDL-C and asthma. Associations unique to Dominicans were demonstrated with a 34% lower odds ratio for each 50 mg/dL higher LDL-C. However, no effect modification was shown between heritage groups and TC or LDL-C so the results should be interpreted cautiously in the setting of multiple comparisons within heritage groups and lipid parameters. Preclinical experiments have shown elevated LDL and apoliproteinE negatively modulate airway hyperreactivity, mucin gene expression and goblet cell hyperplasia which do not support our results of an inverse association [55]. Serum cholesterol levels can decrease during inflammation [56] and the inverse association demonstrated in Latinos/Hispanics may be more reflective of ongoing inflammation in asthma individuals rather than a protective relationship. Overall, our results indicate LDL-C and TC may be an important marker for asthma in Hispanics/Latinos.

The contrast between Hispanic/Latino groups was less pronounced in the relationship between lipid profile and COPD prevalence. No remarkable associations were found in the estimated population. Only Hispanic/Latinos of Central American heritage had 45% greater odds ratio associated with increasing levels of HDL-C; however, these results must also be interpreted with caution because no effect modification was shown between HDL-C and heritage groups. Animal models have shown HDL-C to inhibit tumor necrosis factor-stimulated sphingosine kinase activity and increases ceramide, which causes cellular destruction in emphysema via oxidative stress and apoptosis [57,58]. Barr et al. [9] demonstrated COPD prevalence in HCHS/SOL was not related to Hispanic/Latino heritage after accounting for smoking history and asthma onset prior to age 45. Other characteristics such as effects of migration, geography, and biomass smoke should be addressed in future studies as potential confounders given that one third of U.S. Hispanic/Latinos were born outside the U.S.

The results in this study represent a targeted population of Hispanic/Latino individuals living in four urban communities who are not necessarily representative of the U.S. However, HCHS/SOL’s hybrid design uses probability sampling within pre-selected diverse regions and is superior to the convenience samples typically used in cohort studies. Multiple comparisons in the analyses of multiple lipid parameters was not adjusted for and the associations within heritage groups should be interpreted cautiously. Overlap syndrome of asthma and COPD has become increasingly recognized [38] and the characteristics of this group should be examined further in HCHS/SOL because they represent different characteristics from asthma or COPD alone. In particular, the combined group was similar in age, gender, and U.S. birth to COPD only group but had similarities in prevalences of asthma risk factors to the asthma only group with obesity, current smoking, and atopy. Furthermore, the use of inhalers was higher in this group than either COPD alone or asthma alone. These differences may have affected our results in examining associations between lipid parameters and obstructive lung disease.

## Conclusion

HCHS/SOL is the most comprehensive examination to date of lipid profile and obstructive lung disease in a diverse group of Hispanics/Latinos. We demonstrated a modest inverse association between current asthma and the lipid profile of Hispanics/Latinos, specifically TC and LDL-C. These results highlight some important differences in Hispanics/Latinos and certain serum lipids may be factors or markers of obstructive lung disease.

## Figures and Tables

**Table 1:** Estimated Population Characteristics Overall and by Hispanic/Latino Group (age adjusted).

Estimate (95% Confidence Interval)									
Parameter	Total Population N=14636	Mexican n=5830	Cuban n=2181	Puerto Rican n=2379	Dominican n=1271	Central American n=1567	South American n=972	Other n=436	P value
**Age (year)**	41.2 (40.7–41.7)	38.7 (38.039.4)	46.5 (45.447.6)	43.1 (42.144.2)	39.9 (38.441.3)	39.7 (38.7–40.7)	42.2 (40.6–43.7)	34.5 (32.836.2)	<0.001
**Sex (% Female)**	52.9 (51.8–54.1)	54.8 (52.856.8)	47.2 (45.049.4)	49.8 (46.952.6)	61.4 (57.465.3)	54.0 (50.8–57.3)	55.2 (51.4–59.1)	53.0 (45.560.6)	<0.001
**BMI ≥ 30 (%)**	39.3 (38.0–40.7)	39.6 (36.942.2)	35.7 (33.238.2)	45.5 (42.548.5)	41.5 (37.445.6)	37.3 (34.5–40.1)	30.0 (26.0–33.9)	42.7 (36.249.2)	<0.001
**Education, %**									
**Some H.S.**	32.1 (30.6–33.6)	37.4 (34.640.1)	18.7 (16.620.9)	36.2 (32.739.6)	37.6 (34.141.1)	39.2 (35.7–42.6)	21.3 (17.6–24.9)	24.2 (17.930.5)	<0.001
**H.S. graduate**	28.1 (27.0–29.2)	28.3 (26.530.2)	33.0 (30.235.9)	27.9 (25.530.4)	22.7 (18.826.6)	25.0 (22.1–27.9)	28.3 (24.5–32.1)	19.7 (11.927.4)	<0.001
**> H.S.**	39.8 (38.1–41.5)	34.3 (31.037.6)	48.2 (45.351.2)	35.9 (32.839.0)	39.7 (36.243.3)	35.8 (32.5–39.2)	50.4 (46.1–54.7)	56.1 (48.363.9)	<0.001
**Alcohol %**									
**Never**	18.8 (17.4–20.2)	13.7 (12.115.2)	33.2 (30.536.0)	11.8 (10.0–13.7)	10.5 (8.612.4)	29.9 (26.7–33.1)	20.5 (16.5–24.5)	14.8 (10.019.6)	<0.001
**Former**	29.9 (28.6–31.3)	34.0 (31.836.1)	17.0 (14.919.1)	37.4 (34.839.9)	34.9 (30.739.0)	27.5 (24.6–30.4)	28.4 (24.6–32.2)	26.5 (19.933.1)	<0.001
**Current**	51.3 (49.7–52.8)	52.4 (49.655.1)	49.8 (46.752.8)	50.8 (48.153.5)	54.6 (50.558.8)	42.6 (39.4–45.8)	51.0 (46.6–55.5)	58.7 (51.466.1)	<0.001
**Smoking status %**									
**Never**	63.1 (61.8–64.4)	65.6 (63.367.9)	57.5 (54.560.4)	52.3 (49.355.3)	76.6 (73.180.2)	72.2 (69.1–75.3)	66.8 (62.3–71.2)	56.7 (49.164.3)	<0.001
**Former**	16.4 (15.5–17.2)	18.2 (16.619.8)	15.3 (13.617.0)	14.9 (12.917.0)	12.0 (10.014.0)	14.4 (12.3–16.5)	20.3 (17.1–23.4)	19.5 (14.524.5)	<0.001
**Current**	20.5 (19.3–21.7)	16.2 (14.518.0)	27.2 (24.530.0)	32.8 (30.135.5)	11.4 (8.014.7)	13.4 (10.9–15.8)	13.0 (9.8–16.1)	23.8 (17.330.4)	<0.001
**Pack years (>10%)**	14.4 (13.5–15.3)	8.5 (7.5–9.4)	25.2 (23.027.5)	21.6 (19.423.7)	9.0 (7.5–10.5)	9.2 (7.8–10.7)	9.9 (7.6–12.1)	14.5 (11.217.7)	<0.001
**Statin Medication (%)**	7.8 (7.2–8.3)	7.3 (6.5–8.2)	5.5 (4.3–6.8)	11.6 (10.3–13.0)	9.9 (8.5–11.4)	5.6 (4.6–6.6)	5.7 (4.2–7.2)	9.9 (5.7–14.1)	<0.001
**Non-statin cholesterol medications (%)**	10.5 (9.9–11.1)	10.0 (9.011.0)	8.6 (7.2–10.0)	14.5 (12.916.1)	13.1 (11.214.9)	7.9 (6.8–9.1)	7.8 (5.9–9.7)	12.2 (8.116.3)	<0.001
**Born in US**	22.2 (20.7–23.6)	20.5 (18.522.6)	12.3 (9.914.6)	51.0 (48.353.8)	14.1 (10.717.6)	5.6 (3.1–8.1)	6.6 (4.4–8.9)	48.2 (41.454.9)	<0.001
**Age at immigration %**									
**Age 0–15**	14.0 (13.2–14.9)	13.2 (11.914.6)	11.0 (9.612.5)	22.6 (20.025.1)	15.6 (12.918.4)	10.5 (8.1–12.9)	12.5 (9.4–15.7)	9.8 (3.0–16.7)	<0.001
**Age >15**	63.8 (62.1–65.5)	66.2 (64.068.4)	76.7 (74.079.4)	26.4 (23.429.3)	70.3 (66.274.3)	83.9 (80.6–87.1)	80.9 (77.6–84.1)	42.0 (36.547.5)	<0.001
**Income**									
**<$20,000**	41.7 (39.9–43.4)	37.5 (34.340.6)	44.3 (41.547.2)	44.3 (40.748.0)	50.0 (45.454.7)	46.7 (42.6–50.8)	39.7 (35.5–43.9)	30.5 (24.736.3)	<0.001
**$20,000–50,000**	37.1 (35.7–38.5)	42.4 (40.244.6)	31.8 (29.034.6)	32.0 (28.735.4)	33.7 (29.837.7)	34.1 (30.4–37.8)	41.2 (37.1–45.4)	42.5 (35.149.9)	<0.001
**>50,000**	12.0 (10.3–13.6)	14.9 (12.017.9)	7.6 (5.7–9.6)	14.2 (11.8–16.7)	7.2 (5.0–9.5)	7.0 (4.9–9.0)	10.9 (8.2–13.5)	19.3 (13.125.5)	<0.001
**Others**									
**Not reported**	9.3 (8.5–10.1)	5.2 (4.4–6.0)	16.2 (14.018.4)	9.4 (7.8–10.9)	9.0 (7.0–11.0)	12.3 (10.2–14.4)	8.2 (5.9–10.6)	7.7 (4.3–11.1)	<0.001
**Health insurance (yes/ no) (%)**	50.2 (48.3–52.0)	43.4 (40.646.1)	39.4 (36.342.6)	77.6 (74.880.3)	71.7 (67.775.7)	32.5 (28.4–36.6)	40.8 (36.3–45.4)	60.2 (52.767.7)	<0.001
**Years in US, 10 or more (%)**	71.5 (69.6–73.4)	76.7 (74.479.0)	48.1 (44.651.6)	92.1 (90.294.0)	76.0 (72.079.9)	63.2 (59.2–67.3)	57.8 (53.1–62.5)	88.7 (83.693.8)	<0.001
**TC, mg/dL**	193.6 (192.6194.6)	195.1 (193.5196.8)	196.2 (194.1198.3)	184.8 (182.5187.0)	189.4 (186.6192.3)	200 (197.2–202.8)	198 (195.1200.8)	192.1 (186.6197.7)	<0.001
**HDL-C, mg/dL**	48.8 (48.5–49.2)	48.8 (48.249.3)	48.0 (47.448.6)	48.3 (47.449.2)	50.5 (49.551.5)	48.7 (47.9–49.6)	50.0 (49.0–51.0)	50.8 (49.052.6)	<0.001
**LDL-C, mg/dL**	120 (119.1–120.9)	120.4 (119.0121.8)	123.1 (121.2124.9)	113.4 (111.3115.4)	118.1 (115.5120.8)	124.4 (121.8126.9)	122.8 (120.4125.3)	117.4 (112.3122.4)	<0.001
**TGs, mg/dL**	124.1 (122.4125.7)	129.7 (126.7132.8)	126 (122.8129.3)	115.3 (112.2118.5)	103.9 (100.2107.5)	134.6 (130.4138.8)	125.6 (120.5130.7)	119.8 (111.9127.7)	<0.001
**Oral corticosteroids (%)**	1.3 (1.1–1.6)	1.3 (0.9–1.7)	1.0 (0.5–1.5)	2.7 (1.9–3.4)	0.8 (0.4–1.2)	0.7 (0.3–1.2)	0.8 (0.2–1.4)	1.6 (0.1–3.1)	0.002
**Dyslipidemia (%)**	29.3 (28.3–30.2)	30.5 (28.932.1)	28.9 (26.831.1)	27.3 (25.129.5)	25.7 (22.728.7)	33.5 (30.7–36.3)	29.4 (25.9–32.9)	27.6 (22.532.8)	0.001
**Current asthma (%)**	7.4 (6.7–8.0)	3.3 (2.6–4.0)	7.2 (5.8–8.5)	20.7 (18.522.8)	7.6 (5.9–9.3)	3.5 (2.5–4.5)	3.4 (2.0–4.7)	7.5 (4.7–10.4)	<0.001
Current COPD** **(%) (n=14012)**	3.6 (3.2–4.0)	2.5 (1.9–3.2)	5.1 (4.1–6.1)	5.7 (4.5–7.0)	2.9 (1.7–4.1)	2.4 (1.6–3.1)	2.3(1.1–3.4)	3.0 (1.8–4.2)	<0.001

TC: Total Cholesterol; HDL-C: High Density Lipoprotein Cholesterol; LDL-C: Low Density Lipoprotein Cholesterol; TGs: Triglycerides; COPD: Chronic Obstructive Pulmonary Disease.

* : Values were weighted for survey design and nonresponse and adjusted for age (except for mean ages by Hispanic/Latino group).

** Missing COPD data was accounted for using multiple imputation and inverse probability weighting. Comparisons across Hispanic/Latino heritage groups were performed using the overall Wald test.

**Table 2:** Descriptive Characteristics for All Participants and by Obstructive Airway Disease (age-adjusted).

Estimate (95% Confidence Interval)						
Parameter	Total Population (N=14012)	No Asthma or COPD	Asthma Only	COPD only	Asthma and COPD	P-value
**Age (year)**	41.1 (40.6–41.6)	40.5 (40.0–41.0)	40.3 (39.0–41.7)	57.7 (55.6–59.7)	54.6 (51.7–57.5)	<0.001
**Sex (% Female)**	53.0 (51.7–54.3)	52.4 (51.1–53.7)	72.5 (68.2–76.7)	24.6 (18.8–30.5)	54.4 (43.5–65.3)	<0.001
**Body mass index (kg/m2)**	29.3 (29.2–29.5)	29.2(29.0–29.3)	32.8(32.0–33.5)	26.7(26.0–27.5)	29.7(28.3–31.0)	<0.001
**BMI ≥ 30 (%)**	39.4 (38.0–40.7)	38.4(37.1–39.8)	57.4(53.1–61.7)	24.2(16.8–31.7)	42.3(31.6–53.0)	<0.001
**Education (%)**						
**Some H.S.**	32.4 (30.9–33.9)	31.7(30.1–33.3)	38.1(33.4–42.8)	39.5(32.6–46.5)	39.1(27.6–50.5)	<0.01
**H.S. graduate**	28.1 (27.0–29.3)	28.4(27.2–29.5)	27.6(23.5–31.8)	21.8(16.8–26.7)	28.2(18.3–38.2)	>0.05
**>H.S**	39.5 (37.7–41.2)	39.9(38.2–41.7)	34.3(29.7–38.8)	38.7(31.3–46.1)	32.7(21.5–43.9)	<0.05
**Alcohol (%)**						
**Never**	18.8 (17.4–20.3)	18.9 (17.4–20.3)	20.4 (16.5–24.2)	12.5 (7.5–17.5)	20.5 (12.8–28.3)	<0.05
**Former**	29.9 (28.5–31.3)	29.3 (27.9–30.7)	38.2 (33.7–42.7)	26.4 (19.8–32.9)	37.6 (25.9–49.3)	<0.001
**Current**	51.3 (49.7–52.9)	51.8 (50.2–53.5)	41.5 (37.0–46.0)	61.2 (54.1–68.2)	41.9 (31.7–52.1)	<0.001
**Smoking status (%)**						
**Never**	62.9 (61.6–64.3)	64.3 (62.9–65.7)	56.3 (52.2–60.5)	35.3 (28.0–42.5)	52.5 (41.4–63.6)	<0.001
**Former**	16.4 (15.5–17.2)	16.2 (15.3–17.1)	18.1 (14.0–22.2)	19.4 (13.5–25.3)	14.6 (5.9–23.2)	>0.05
**Current**	20.7 (19.5–21.9)	19.5 (18.2–20.8)	25.6 (21.8–29.3)	45.3 (38.8–51.9)	33.0 (24.9–41.0)	<0.001
**Pack years, above 10, %**	14.3 (13.4–15.3)	13.0 (12.1–13.9)	17.9 (14.9–20.8)	46.2 (39.2–53.1)	32.2 (21.7–42.7)	<0.001
**Statin Medication (%)**	7.9 (7.3–8.5)	7.6 (7.0–8.3)	13.1 (10.8–15.5)	3.7 (−1.2–8.6)	10.7 (2.5–18.8)	<0.001
**Non-statin cholesterol medications (%)**	10.7 (10.0–11.3)	10.2 (9.5–10.9)	18.5 (15.7–21.3)	5.7 (0.8–10.7)	11.9 (2.5–21.3)	<0.001
**Born in US**	22.4 (20.9–23.9)	20.9 (19.4–22.4)	39.7 (35.4–44.1)	28.5 (22.9–34.2)	30.5 (23.9–37.2)	<0.001
**Age at immigration (%)**						
**Age (0–15)**	13.9 (13.0–14.8)	13.6 (12.6–14.6)	16.0 (12.9–19.1)	12.9 (10.0–15.9)	27.3 (17.1–37.6)	<0.05
**Age (>15)**	63.7 (62.0–65.5)	65.5 (63.8–67.3)	44.3 (40.5–48.1)	58.5 (52.6–64.4)	42.1 (30.7–53.6)	<0.001
**Health insurance (yes/no) (%)**	50.6 (48.7–52.5)	48.8 (46.9–50.7)	72.7 (68.5–77.0)	51.0 (43.3–58.7)	67.5 (57.5–77.4)	<0.001
**Years in US, 10 or more (%)**	71.5 (69.6–73.5)	70.7 (68.7–72.7)	83.7 (80.1–87.3)	68.8 (62.4–75.2)	74.7 (65.5–84.0)	<0.001
**Income**						
**<20,000**	41.3 (39.5–43.1)	40.1(38.3–42.0)	55.6 (50.9–60.2)	42.8 (35.0–50.6)	52.1 (41.7–62.5)	<0.001
**20,000–50,000**	37.0 (35.6–38.4)	38.4 (36.9–39.8)	22.2 (18.5–25.9)	31.5 (26.2–36.9)	23.9 (15.7–32.1)	<0.001
**>50,000**	11.9 (10.3–13.5)	12.2 (10.5–13.8)	9.0 (6.5–11.6)	11.7 (5.5–17.8)	8.7 (0.7–16.7)	>0.05
**Not reported**	9.7 (8.9–10.6)	9.3 (8.4–10.2)	13.2 (10.2–16.2)	14.0 (9.1–18.9)	15.3 (6.8–23.8)	<0.01
**TC (mg/dL)**	193.4 (192.3–194.4)	194.1 (193.0–195.3)	187.1 (183.8–190.4)	188.3 (180.8–195.8)	177.9 (170.0–185.8)	<0.001
**HDL-C (mg/dL)**	48.8 (48.5–49.2)	48.9(48.5–49.2)	48.7(47.6–49.9)	47.8(46.1–49.6)	49.8(46.9–52.8)	>0.05
**LDL-C (mg/dL)**	119.8 (118.8–120.7)	120.4 (119.4–121.4)	114.3 (111.5–117.2)	117.6 (111.3–123.8)	107.5 (100.6–114.3)	<0.001
**TGs (mg/dL)**	123.7 (122.0–125.3)	124.4 (122.7–126.2)	120.3 (114.8–125.7)	114.4 (106.6–122.2)	103.1 (91.2–115.1)	<0.001
**Dyslipidemia (%)**	29.2 (28.3–30.2)	29.6 (28.5–30.7)	29.4 (26.0–32.8)	19.8 (13.7–25.9)	19.4 (8.8–30.0)	<0.01
**Oral corticosteroids (%)**	1.3 (1.1–1.5)	0.6 (0.5–0.8)	8.8 (6.2–11.3)	1.5 (−0.3–3.4)	12.0 (6.0–18.0)	<0.001
**Use of Asthma medications (%)**	6.4 (5.7–7.0)	0	83.0 (79.4–86.6)	0	94.1 (90.0–98.3)	<0.001
**Use of COPD medications (%)**	1.8 (1.5–2.2)	0.6 (0.4–0.8)	14.7 (12.0–17.3)	2.3 (0.3–4.4)	31.8 (22.3–41.3)	<0.001
**Atopy (%)**	12.9 (12.0–13.8)	11.4 (10.4–12.3)	32.0 (27.7–36.3)	7.7 (3.3–12.2)	37.0 (25.8–48.1)	<0.001

* Values were weighted for survey design and nonresponse and adjusted for age (except for mean ages by Hispanic/Latino group. Missing COPD data was accounted for using multiple imputation and inverse probability weighting.

Comparisons across obstructive airway disease groups were performed using the overall Wald test.

**Table 3:** Age-statin use-adjusted Mean Serum Lipid Levels among All Participants With and Without Asthma and by Hispanic/Latino Heritage Groups.

Estimate (95% Confidence Interval)								
Parameter	Total Population (N=14636)	Mexican (n=5830)	Cuban (n=2181)	Puerto Rican (n=2379)	Dominican (n=1271)	Central American (n=1567)	South American (n=972)	Other (N=436)
**TC (mg/dL)**								
**Asthma: Yes**	187.3 (184.3–190.3)	191.4 (186.4196.3)	192.9 (185.3200.4)	182.7 (178.1187.3)	182 (175.0188.9)	197.3 (184.5–210.0)	186.2 (169.9–202.6)	193.8 (179.6207.9)
**Asthma: No**	194.1 (193.1–195.1)	195.1 (193.5196.8)	195.8 (193.8197.9)	186.4 (183.9189.0)	190.6 (187.5193.7)	199.6 (196.7–202.4)	197.8 (194.9200.7)	192.7 (187.1198.3)
**HDL-C, mg/dL**								
**Asthma: Yes**	49.0 (48.0–50.0)	49.2 (46.851.6)	49.0 (46.7–51.3)	48.0 (46.5–49.6)	50.5 (47.4–53.6)	52.3 (47.9–56.8)	47.6 (43.2–52.1)	50.9 (45.456.4)
**Asthma: No**	48.8 (48.5–49.2)	48.8 (48.249.3)	47.9 (47.2–48.5)	48.4 (47.5–49.4)	50.6 (49.5–51.6)	48.6 (47.7–49.4)	50.0 (49.0–51.0)	50.8 (48.952.8)
**LDL-C, mg/dL**								
**Asthma: Yes**	114.9 (112.2–117.5)	117.9 (112.5123.4)	118.8 (112.6124.9)	111.8 (107.8115.8)	110.8 (104.6117.0)	119.4 (109.3–129.6)	112.9 (99.3–126.5)	120.5 (105.7135.2)
**Asthma: No**	120.3 (119.5–121.2)	120.4 (118.9121.8)	122.7 (120.9124.5)	114.9 (112.7117.2)	119.3 (116.5122.1)	124 (121.4–126.6)	122.6 (120.2–125.0)	117.8 (112.7122.9)
**TGs, mg/dL**								
**Asthma: Yes**	117.4 (113.1–121.8)	121.3 (111.4131.2)	125.3 (114.9135.7)	114.3 (107.8120.9)	103 (94.5–111.5)	127.3 (107.3–147.3)	128.7 (91.9–165.5)	112 (96.9127.2)
**Asthma: No**	124.6 (122.8–126.3)	130 (126.8133.2)	126.2 (122.8129.6)	115.2 (111.6118.7)	103.7 (99.7107.7)	135 (130.8–139.3)	125.7 (120.5–130.8)	120.2 (111.7128.8)

**Table 4:** Association between serum lipids and asthma for all participants and by Hispanic/Latino heritage groups.

Odds Ratio (95% Confidence Interval)								
Parameter	Total Population (N=14636)	Mexican (n=5830)	Cuban (n=2181)	Puerto Rican (n=2379)	Dominican (n=1271)	Central American (n=1567)	South American (n=972)	Other (N=436)
**TC (50mg/dL)**								
Unadjusted OR*	0.83 (0.75–0.92)	0.87 (0.721.05)	0.89 (0.731.09)	0.89 (0.75–1.05)	0.78 (0.61–0.99)	1.02 (0.67–1.53)	0.76 (0.45–1.30)	1.08 (0.631.86)
**Age-adjusted OR**†	0.78 (0.70–0.86)	0.87 (0.731.05)	0.88 (0.701.10)	0.87 (0.74–1.03)	0.73 (0.56–0.95)	0.93 (0.62–1.38)	0.62 (0.33–1.17)	1.13 (0.642.00)
**Fully Adjusted OR**‡	0.87 (0.78–0.97)	1.05 (0.861.29)	0.86 (0.671.10)	0.87 (0.73–1.04)	0.71 (0.53–0.95)	0.99 (0.66–1.49)	0.64 (0.32–1.31)	1.16 (0.612.20)
**HDL-C (10mg/dL)**								
Unadjusted OR*	1.01 (0.95–1.08)	1.00 (0.861.17)	1.10 (0.951.26)	0.97 (0.87–1.09)	0.98 (0.80–1.20)	1.23 (1.03–1.49)	0.88 (0.63–1.23)	0.98 (0.701.37)
**Age-adjusted OR**†	1.00 (0.94–1.07)	1.01 (0.861.18)	1.10 (0.951.27)	0.97 (0.87–1.08)	0.98 (0.80–1.20)	1.22 (1.02–1.45)	0.87 (0.63–1.20)	0.98 (0.701.37)
**Fully Adjusted OR**‡	1.02 (0.94–1.10)	1.01 (0.861.20)	1.08 (0.921.29)	0.97 (0.85–1.11)	1.00 (0.78–1.27)	1.15 (0.92–1.44)	0.82 (0.60–1.14)	1.06 (0.721.57)
**LDL-C (50mg/dL)**								
Unadjusted OR*	0.80 (0.72–0.90)	0.88 (0.691.14)	0.82 (0.651.03)	0.86 (0.71–1.04)	0.71 (0.53–0.94)	0.90 (0.58–1.41)	0.69 (0.37–1.32)	1.20 (0.612.33)
**Age-adjusted OR**†	0.76 (0.68–0.86)	0.89 (0.691.16)	0.81 (0.631.03)	0.85 (0.70–1.03)	0.67 (0.50–0.91)	0.83 (0.53–1.29)	0.58 (0.29–1.18)	1.27 (0.632.55)
**Fully Adjusted OR**‡	0.86 (0.76–0.97)	1.14 (0.861.51)	0.80 (0.621.02)	0.87 (0.72–1.06)	0.66 (0.48–0.91)	0.90 (0.58–1.42)	0.62 (0.28–1.39)	1.31 (0.592.91)
**TGs (50mg/dL)**								
Unadjusted OR*	0.94 (0.89–1.00)	0.91 (0.791.05)	0.98 (0.871.10)	1.00 (0.92–1.10)	1.03 (0.89–1.20)	0.95 (0.75–1.21)	1.07 (0.77–1.49)	0.89 (0.671.18)
**Age-adjusted OR**†	0.92 (0.87–0.98)	0.92 (0.791.05)	0.98 (0.861.11)	1.00 (0.92–1.09)	1.02 (0.86–1.20)	0.90 (0.71–1.13)	1.02 (0.68–1.51)	0.89 (0.671.19)
**Fully Adjusted OR**‡	0.94 (0.88–1.00)	0.90 (0.771.06)	0.98 (0.861.12)	0.96 (0.86–1.07)	0.95 (0.78–1.15)	0.98 (0.74–1.29)	0.97 (0.65–1.44)	0.79 (0.511.22)

* From unadjusted model;

† From age-adjusted model;

‡ Fully adjusted model including age, gender, ethnicity, cigarette smoking, alcohol intake, BMI, all lipid/cholesterol-lowering medications, health insurance , age at immigration

**Table 5:** Age-statin use-adjusted mean serum lipid levels among all participants with and without copd and by Hispanic/Latino heritage groups.

Estimate (95% Confidence Interval)								
Parameter	Total Population (N=14012)	Mexican (n=5651)	Cuban (n=2116)	Puerto Rican (n=2204)	Dominican (n=1178)	Central American (n=1502)	South American (n=941)	Other (N=420)
**TC (mg/dL)**								
**COPD: Yes**	191.3 (181.7–201.0)	198.8 (181.9215.6)	195.2 (184.0206.4)	183.9 (172.8195.0)	177.9 (158.5197.2)	197.6 (185.1–210.1)	201.5 (178.6–224.5)	181.4 (160.7202.0)
**COPD: No**	193.8 (192.7–194.8)	195.1 (193.4196.8)	196.2 (194.1198.3)	185.6 (183.1188.1)	189.7 (186.5192.9)	199.5 (196.7–202.3)	197.4 (194.4–200.4)	193 (187.6198.3)
**HDL-C (mg/dL)**								
**COPD: Yes**	46.5 (44.1–48.8)	47.7 (43.851.5)	45.8 (42.748.9)	46.4 (43.2–49.5)	44.9 (41.2–48.6)	49.8 (41.9–57.8)	47.6 (37.7–57.6)	37.8 (31.5–44.2)
**COPD: No**	48.9 (48.5–49.2)	48.8 (48.249.3)	48.0 (47.448.6)	48.2 (47.4–49.1)	50.8 (49.7–51.9)	48.5 (47.7–49.3)	49.8 (48.8–50.8)	51.0 (49.1–52.9)
**LDL-C (mg/dL)**								
**COPD: Yes**	119.9 (111.8–127.9)	125.1 (111.8138.3)	122.6 (112.6132.6)	114.8 (104.5125.1)	111.9 (94.6129.2)	117.1 (106.9–127.3)	131.4 (109.7–153.0)	114.4 (94.8134.0)
**COPD: No**	120.1 (119.1–121.0)	120.3 (118.8121.8)	123 (121.1124.9)	114.2 (112.0116.5)	118.2 (115.3121.2)	124.2 (121.7–126.8)	122.2 (119.7–124.8)	118.1 (113.2123.0)
**TGs (mg/dL)**								
**COPD: Yes**	124.5 (113.2–135.8)	129.6 (115.9143.3)	133.8 (115.8151.9)	113.3 (98.6–127.9)	105.3 (84.8125.7)	153.5 (115.4–191.6)	112.1 (88.0–136.3)	144.9 (98.3191.4)
**COPD: No**	124.2 (122.5–125.9)	130 (126.9133.2)	126.1 (122.7129.5)	115.6 (112.1119.0)	103.7 (99.6107.8)	133.9 (129.7–138.0)	126.8 (121.6–132.0)	119.6 (110.9128.3)

**Table 6:** Association between serum lipids and COPD for all participants and by Hispanic/Latino heritage groups.

Odds Ratio (95% Confidence Interval)								
Parameter	Total Population (N=14012)	Mexican (n=5651)	Cuban (n=2116)	Puerto Rican (n=2204)	Dominican (n=1178)	Central American (n=1502)	South American (n=941)	Other (N=420)
**TC (50mg/dL)**								
Unadjusted OR*	1.21 (1.04–1.40)	1.39 (0.95–2.04)	1.13 (0.90–1.42)	1.19 (0.90–1.57)	0.89 (0.49–1.60)	1.21 (0.93–1.59)	1.39 (0.79–2.42)	1.07 (0.56–2.04)
**Age-adjusted OR**†	0.89 (0.75–1.04)	1.07 (0.68–1.69)	0.89 (0.69–1.14)	0.91 (0.67–1.22)	0.66 (0.35–1.24)	0.88 (0.63–1.24)	0.97 (0.51–1.87)	0.68 (0.32–1.46)
**Fully Adjusted OR**‡	0.93 (0.78–1.11)	1.10 (0.66–1.82)	0.87 (0.66–1.16)	0.92 (0.68–1.24)	0.80 (0.46–1.42)	0.93 (0.60–1.44)	1.26 (0.69–2.31)	0.26 (0.03–2.12)
**HDL-C (10mg/dL)**								
Unadjusted OR*	1.03 (0.93–1.13)	1.07 (0.88–1.30)	1.08 (0.92–1.26)	1.05 (0.89–1.23)	0.76 (0.52–1.12)	1.24 (0.88–1.76)	1.07 (0.53–2.13)	0.42 (0.21–0.82)
**Age-adjusted OR**†	0.96 (0.87–1.06)	1.03 (0.84–1.27)	0.99 (0.83–1.18)	0.93 (0.79–1.09)	0.72 (0.49–1.07)	1.18 (0.86–1.61)	0.92 (0.49–1.72)	0.36 (0.17–0.77)
**Fully Adjusted OR**‡	1.02 (0.92–1.13)	1.07 (0.82–1.39)	1.00 (0.85–1.19)	0.96 (0.80–1.16)	0.75 (0.51–1.09)	1.45 (1.17–1.79)	1.22 (0.64–2.33)	0.41 (0.12–1.41)
**LDL-C (50mg/dL)**								
Unadjusted OR*	1.21 (1.02–1.43)	1.43 (0.96–2.14)	1.08 (0.84–1.41)	1.21 (0.86–1.70)	0.93 (0.49–1.77)	0.93 (0.65–1.33)	1.61 (0.81–3.19)	1.24 (0.61–2.51)
**Age-adjusted OR**†	0.93 (0.78–1.11)	1.14 (0.74–1.76)	0.89 (0.68–1.16)	0.99 (0.69–1.40)	0.74 (0.39–1.40)	0.70 (0.48–1.03)	1.22 (0.57–2.61)	0.82 (0.37–1.83)
**Fully Adjusted OR**‡	0.95 (0.78–1.15)	1.14 (0.70–1.86)	0.85 (0.62–1.16)	1.00 (0.71–1.40)	0.91 (0.50–1.67)	0.69 (0.43–1.10)	1.55 (0.78–3.09)	0.25 (0.01–4.23)
**TGs (50mg/dL)**								
Unadjusted OR*	1.07 (1.00–1.15)	1.06 (0.93–1.21)	1.07 (0.95–1.22)	1.01 (0.89–1.16)	1.12 (0.85–1.46)	1.26 (0.97–1.64)	0.90 (0.69–1.17)	1.45 (0.98–2.16)
**Age-adjusted OR**†	0.91 (0.83–0.99)	0.91 (0.77–1.07)	0.95 (0.81–1.12)	0.88 (0.74–1.05)	0.89 (0.64–1.24)	1.07 (0.73–1.58)	0.67 (0.42–1.06)	1.29 (0.81–2.07)
**Fully Adjusted OR**‡	0.92 (0.84–1.00)	0.93 (0.79–1.09)	0.98 (0.81–1.18)	0.88 (0.73–1.06)	0.92 (0.65–1.32)	0.99 (0.74–1.31)	0.60 (0.38–0.96)	1.17 (0.69–1.98)

* From unadjusted model;

† From age-adjusted model;

‡ Fully adjusted model including age, gender, ethnicity, cigarette smoking, alcohol intake, BMI, all lipid/cholesterollowering medications, health insurance status, age at immigration, asthma onset before age 45 (yes/no); .Missing COPD data was accounted for using multiple imputation and inverse probability weighting.
